# Delabeling penicillin allergies in plastic and reconstructive surgery: Cefazolin as the cornerstone for antibiotic prophylaxis^✰^

**DOI:** 10.1016/j.jpra.2026.07.025

**Published:** 2026-07-23

**Authors:** Natalia Mejia, Sajni Parikh, Aryan Gupta, Isabella Ho, Alexander Chang, Gabriel De La Cruz, Adam Walchak, Sameer A. Patel

**Affiliations:** a Division of Plastic and Reconstructive Surgery, Fox Chase Cancer Center/Temple University, Philadelphia, PA, USA; b Division of Plastic and Reconstructive Surgery, Temple University Hospital, Philadelphia, PA, USA; c Lewis Katz School of Medicine, Temple University, Philadelphia, PA, USA; d Universidad Cientifica del Sur, Lima, Peru

**Keywords:** Cefazolin, Surgical antibiotic prophylaxis, Cross-reactivity, Β-lactam allergy, Surgical site infection

## Abstract

Cefazolin is frequently used as surgical prophylaxis due to its efficacy in preventing surgical site infections (SSIs). However, in patients with reported history of allergy to penicillin, there is a disproportionate use of alternative antibiotic options for prophylaxis. This practice is associated with increased rates of surgical site infection, secondary infections, longer hospital stays, and higher healthcare costs. The use of these alternative options is based on several misconceptions on antibiotic safety; penicillin allergies are frequently over-recorded in the medical record, with <10% of reported patients being truly allergic to penicillin. Even in the case of true allergy, the risk of reactivity to cefazolin is extremely low due to its unique structure, as compared to penicillin/penicillin-like β-lactams. Improved patient outcomes may be achieved through increased clinician education on the safety of cefazolin for surgical prophylaxis, as well as thorough understanding of the rare cases where its use is truly contraindicated. Similarly, there are currently limited guidelines on the selection of prophylactic agents in the field of plastic surgery. Given the clinical evidence, cefazolin should be considered first in plastic surgery cases where antibiotic prophylaxis is indicated.

## Introduction

Approximately 10% of patients in the United States carry a penicillin allergy label; however, >90% of these individuals do not have a true allergy when formally evaluated.^1^ These allergy labels are often assigned during childhood and can incorrectly persist in patient charts due to a number of reasons, including intolerance, skin reactions not secondary to medication hypersensitivity, viral exanthem, or drug-infection interactions.^2^ Over time, many reported allergies resolve, with studies demonstrating that 60% to 80% of patients lose reactivity after 5 to 10 years.^3^

In surgical practice, inaccurate penicillin allergy labels frequently lead to the use of alternative antibiotic prophylaxis, contributing to suboptimal antimicrobial coverage and increased microbial resistance.^4^ In plastic and reconstructive surgery, the first-line antibiotic prophylaxis for most procedures is a first-generation cephalosporin, Ancef® (cefazolin; GlaxoSmithKline, Philadelphia, PA, USA). Due to its unique molecular structure, cefazolin has a low risk for cross-reactivity when compared to both penicillin and other cephalosporin antibiotics, making it a safe option even for patients with penicillin allergies.^5^^,^^6^

Despite this evidence, avoidance of β-lactam antibiotics remains common in patients with penicillin allergy labels. This practice is largely driven by misconceptions regarding cross-reactivity and failure to appropriately stratify allergy severity. As a result, many patients receive second-line prophylactic agents unnecessarily, which has been associated with higher rates of surgical site infection, increased healthcare utilization, and greater cost burden.^4^^,^^7^ This persistent misconception of cefazolin’s cross-reactivity with penicillin leads to suboptimal antibiotic selection, increasing the risk of surgical site infections, readmissions, and healthcare costs.^8^

Addressing misconceptions surrounding penicillin allergy labeling is critical to optimizing perioperative antibiotic selection in plastic and reconstructive surgery. A clearer understanding of when cefazolin can be safely administered, and when alternative agents are truly indicated, may optimize perioperative outcomes in plastic and reconstructive surgery.

## Antibiotic prophylaxis in patients with penicillin allergy label: evidence supporting first-generation cephalosporins

Understanding the mechanism of β-lactam hypersensitivity is essential to contextualize the true risk of cross-reactivity between penicillins and cephalosporins. The prevalence of true IgE-mediated penicillin allergy has declined over time, likely secondary to decreased use of parenteral penicillins and the relative rarity of anaphylactic reactions to oral amoxicillin.^9^ In contrast, although cephalosporin allergy is uncommon, with approximately 2% of the population reporting a reaction, it is increasingly being recognized as a cause of perioperative anaphylaxis.^9^

**Fig. 1:**
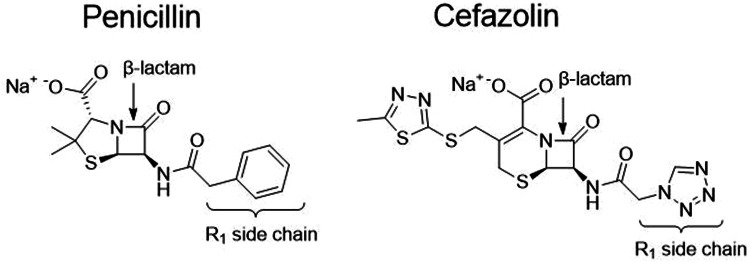
Molecular structures for Penicillin and Cefazolin *All structures were created by the authors using ChemDraw. The chemical structures of penicillin and cefazolin are shown with their respective R1 side chains indicated by brackets. The structural distinction between these side chains is thought to account for the low cross-reactivity observed between the two antibiotics.

Allergic reactions to β-lactam are mediated by haptenation, a process in which the β-lactam ring of the penicillin skeleton opens spontaneously and binds covalently to lysine residues on host proteins, forming penicilloyl-protein complexes. These complexes act as antigens, triggering T-cell activation and B-cell production of drug-specific IgE antibodies.^3^^,^^10^^,^^11^ Among patients with a history of immediate hypersensitivity reaction to penicillin, cross-reactivity with cephalosporins has been historically reported to be as high as 40% in early studies, although more recent evidence suggests substantially lower rates.^6^ However, this risk is largely confined to cephalosporins that share similar side chains to penicillin. While both penicillins and cephalosporins share a β-lactam ring, cefazolin possesses a unique molecular structure through its R1 group (Fig. 1). This side chain plays a critical role in antigen recognition and likely accounts for cefazolin’s minimal cross-reactivity with penicillin.^1^^,^^9^^,^^12^ Therefore, many patients with a history of IgE-mediated reactions to penicillin–including anaphylaxis–may still safely receive cefazolin in the absence of severe delayed hypersensitivity reactions.^4^

Only those patients with a history of true severe, delayed hypersensitivity reactions to penicillin should receive alternative prophylaxis in place of cefazolin. Such reactions include SCARs, encompassing Stevens–Johnson syndrome (SJS), toxic epidermal necrolysis (TEN), DRESS (Drug Reaction with Eosinophilia and Systemic Symptoms), and AGEP (Acute Generalized Exanthematous Pustulosis).^13^^,^^14^ Other less common mechanisms within the SCAR category include type II and type III hypersensitivity reactions, which involve antibody- or complement-mediated injury. Type II reactions may cause conditions such as hemolytic anemia, hepatitis and nephritis, while type III reactions can lead to immune complex mediated vasculitis and serum sickness.^15^ Although these reactions are rare, their associated high mortality underscores their clinical significance. Toxic epidermal necrolysis has an incidence of 0.4–1.2 cases per million person-years, with reported mortality rates of 20–30%.^15^^,^^16^

It is vital to distinguish SCARs from IgE-mediated reactions to penicillin, such as urticaria, angioedema, or anaphylaxis. Although these reactions are immediate and potentially severe, they are not contraindications to cefazolin, as a penicillin-allergic patient would not cross-react to cefazolin’s R1 side chain.^17^ Therefore, even patients with a type I IgE-mediated hypersensitivity reaction to penicillins (including anaphylaxis and rash) may still be candidates for cefazolin use.

Multiple studies support the safety of cefazolin use in penicillin-allergic patients, reinforcing its selectiveness. In a meta-analysis examining patients with reported history of penicillin allergy, out of 9225 patients that underwent direct penicillin challenge, only 438 experienced a reaction (3.5% overall reaction rate, 2.2% for adults and 6.6% for children). Out of these 438, only 5 experienced severe reactions, with 4 being pediatric patients (3 immediate anaphylaxis and 1 delayed IgE-mediated rash and fever). The only adult severe reaction was a delayed onset AKI. There were no recorded incidents of SCARs or fatal reactions. Through its analysis of 56 prior studies, this paper not only highlights the rarity of truly penicillin-allergic patients, it provides evidence on the safety of direct challenge testing.^18^

In a separate cohort of 66 patients with perioperative allergic reactions, 19 patients tested positive for cefazolin on skin-testing, and in the absence of any other identifiable causing agent, cefazolin was determined to be responsible for the perioperative reaction; none of the 19 patients reacted to alternative β-lactams, and 11 tolerated other cephalosporins.^19^ Similarly, in a large cohort study with 452 patients labeled as penicillin-allergic, none had a positive skin test to cefazolin or ceftriaxone.^20^ This supports the concept that cefazolin’s unique R1 side chain limits cross-reactivity with other β-lactams, including within the cephalosporins class.^9^^,^^14^^,^^18^

## Current practice patterns in plastic surgery

Antibiotic prophylaxis is meant to mitigate possible bacterial contamination at the time of surgical intervention, thereby preventing surgical site infections. SSIs are defined as incisional or organ space infections occurring within the first 30 days of surgical intervention, or within 90 days if the case involves prosthesis implantation.^21^^,^^22^ Centers for Disease Control and Prevention Guidelines for the Prevention of Surgical Site Infection recommend administration of a single dose antibiotic that targets the skin flora, commonly colonized by gram positive microbes.^12^ (Table 1. Recommended Perioperative Antibiotic Prophylaxis for Plastic and Reconstructive Surgery)

**Table 1:** Recommended Perioperative Antibiotic Prophylaxis for Plastic and Reconstructive Surgery – Fox Chase Cancer Center Protocol.

Surgical Category	Patients Without True β-Lactam Contraindication^a^	Patients With True β-Lactam Contraindication^b^
Clean procedures without risk factors	No antibiotic prophylaxis	No antibiotic prophylaxis
Clean procedures with risk factors	Cefazolin 2 g IV (<120 kg) or 3 g IV (≥120 kg), administered within 60 min of incision	Clindamycin 900 mg IV, administered within 60 min of incision
Oral cavity / head and neck procedures	Cefazolin 2 g IV (<120 kg) or 3 g IV (≥120 kg)	Clindamycin 900 mg IV
Gastroduodenal / hernia repair / non-obstructed small intestine	Cefazolin 2 g IV (<120 kg) or 3 g IV (≥120 kg)	Clindamycin 900 mg IV and Ciprofloxacin 400 mg IV
Obstructed small intestine / colorectal surgery	Ertapenem 1 g IV	Ciprofloxacin 400 mg IV and Metronidazole 500 mg IV
Mastectomy with or without reconstruction	Cefazolin 2 g IV (<120 kg) or 3 g IV (≥120 kg)	Clindamycin 900 mg IV

a Includes patients without a penicillin allergy or with non-severe reactions. IgE-mediated reactions alone (urticaria, angioedema, anaphylaxis) are not absolute contraindications to cefazolin.

b True β-lactam contraindications include severe delayed hypersensitivity reactions and type II or III hypersensitivity reactions. Clean procedures were defined as CDC Class I surgical wounds involving uninfected skin and soft tissue without entry into the respiratory, gastrointestinal, genitourinary, or biliary tracts. Recommended intraoperative redosing intervals are every 4 h for cefazolin and every 6 h for clindamycin. No routine intraoperative redosing is recommended for ciprofloxacin. Postoperative antibiotic prophylaxis, when indicated, should be limited to <24 h. Recommended regimens include cefazolin 1 g IV every 8 h or clindamycin 600 mg IV every 8 h. Routine postoperative continuation of ciprofloxacin-based or metronidazole-based prophylaxis is not recommended.

Plastic and reconstructive surgery encompasses a wide range of procedures, ranging from oncoplastic, head and neck, breast surgery, melanoma and skin cancer, and aesthetics. However, to date, there are no field-specific clinical guidelines addressing perioperative antibiotic prophylaxis. Clinical practice guidelines for antimicrobial prophylaxis across all surgical specialities suggests that elective clean surgeries are not required to have prophylaxis for cases in which only skin and soft tissue are involved.^23^ However, providers may still choose to proceed with antibiotic prophylaxis in these cases in order to reduce risk of SSI. For both clean surgeries (i.e. breast reduction and mastectomy) and for procedures with elevated infection risk (i.e. prosthesis placement), administration of single dose intravenous cefazolin is recommended within 1 hour of incision.^12^^,^^23^ For patients with a high-risk penicillin or cephalosporin allergy (e.g., anaphylaxis or severe cutaneous adverse reactions (SCARSs)), a single dose of clindamycin is recommended as an alternative.^9^

Limited evidence exists to guide perioperative antibiotic prophylaxis in plastic and reconstructive surgery, with current recommendations largely summarized in the Consensus Conference Statement from the American Association of Plastic Surgeons.^24^ These guidelines suggest no clear benefit of routine antibiotic prophylaxis in clean surgeries, with breast augmentation being the only exception. For contaminated procedures (class II-IV), prophylaxis is strongly recommended and should be limited to a single perioperative dose, with extension beyond 24 h having no additional benefit.^24^ However, while the consensus suggests switching antibiotic agents in patients with reported allergy or intolerance, it offers no further clarifications.

We present a concise overview of our institution’s preoperative prophylaxis recommendations for plastic and reconstructive procedures (Table 1), which reflect our prior discussion showing that patients labeled as penicillin-allergic can still safely receive cefazolin. Increasing provider education remains essential to reinforce the safety of cefazolin as perioperative prophylaxis, clarifying that penicillin allergy has a low risk of cross-reactivity with cefazolin.

## Misconceptions and pitfalls in antibiotic prophylaxis

Misconceptions regarding cross-reactivity between penicillins and cephalosporins continue to drive the unnecessary use of second-line prophylactic agents in surgical practice. Commonly used alternative antibiotics are clindamycin, fluoroquinolones, and vancomycin, despite their inferior coverage of typical skin flora compared to first-generation cephalosporins. The use of these agents is not benign and has been consistently associated with increased rates of surgical site infection (SSI), antimicrobial resistance, a greater risk of Clostridioides difficile infection and increased healthcare utilization.^4^^,^^25^, ^26^, ^27^

Beyond knowledge gaps, perioperative antibiotic selection can be also influenced by interpersonal and systems-level factors. In practice, anesthesia teams may feel pressure to avoid disagreement in the operating room or may prefer second-line agents to prevent perceived conflict with the surgical team when a penicillin allergy label is present, reflecting recognized behavioral and workflow barriers in perioperative antimicrobial stewardship.^28^^,^^29^ This dynamic can lead to automatic substitution of cefazolin despite evidence supporting its safety in most labeled patients.^4^^,^^9^ Greater transparency in perioperative decision-making, with explicit discussion of the patient’s allergy history, surgical goals, and evidence-based prophylaxis options, is essential.^28^^,^^30^ Establishing shared institutional protocols between surgeons, anesthesiologists, pharmacists, and nursing teams may reduce unnecessary avoidance of cefazolin and improve patient outcomes.^9^^,^^28^, ^29^, ^30^

In a large retrospective cohort study, Kimberly et al. demonstrated that patients labeled as penicillin-allergic at Massachusetts General Hospital had a 1.95-fold higher odds of developing a surgical site infection across a range of procedures, including arthroplasty, hysterectomy, colon surgery, and coronary artery bypass grafting. This increased risk was largely attributable to differences in antibiotic selection rather than allergy status itself, as penicillin-allergic patients received cefazolin far less frequently than non-allergic patients (12%vs. 92%) and were more commonly treated with alternative agents such as clindamycin (49%vs. 3%), vancomycin (35%vs. 3%), and gentamicin (24%vs. 3%).^25^

Microbiologic data further support the inferiority of second-line agents in surgical prophylaxis. In an analysis of national antibiograms, cefazolin (or oxacillin, used as a cefazolin surrogate) demonstrated susceptibility against 99.2 ± 4.8% of methicillin-sensitive *Staphylococcus aureus* (MSSA) isolates, compared to 75.8 ± 8.4% for clindamycin.^31^ Given the high prevalence of MSSA colonization in the general public, and the higher risk of SSI associated with MSSA carriage, clindamycin’s reduced coverage of normal skin flora correlates with the increased risk for SSI in patients given clindamycin prophylaxis.^25^ Similarly, another study published in *JAMA Open*, using data from the Swissnoso SSI surveillance system across 175 hospitals, found that adult patients undergoing major surgery who received surgical antibiotic prophylaxis (SAP) within 120 min prior to incision had significantly higher SSI rates when given non–β-lactam SAP compared with β-lactam SAP (adjusted odds ratio [aOR], 1.78; 95% CI, 1.59–1.99; P < .001). This increased risk with non–β-lactam SAP was observed across all types of surgical procedures.^32^

Although increased SSI rates among patients labeled as penicillin allergic have been demonstrated across different surgical specialties, literature within plastic and reconstructive surgery remains limited. A small retrospective chart review published by The Canadian Society of Plastic Surgeons looked at the antibiotic prophylaxis for 457 body contouring cases among patients labeled as penicillin allergic. The study reported β-lactam allergic patients who received cefazolin as prophylaxis experienced no anaphylactic reactions. Additionally, among those who received second line prophylaxis with Clindamycin, three developed an SSI, compared with only one SSI in the cefazolin group. Larger studies are needed to better define the risk associated with second-line antibiotic prophylaxis for SSI in plastic surgery.^7^ Despite the limited evidence in the field, it is reasonable to infer that, given the less appropriate bacterial coverage of second-line agents, these regimens are less effective than cefazolin across multiple surgical procedures, including plastic and reconstructive surgeries.

Beyond the increased risk of SSI, the use of alternative prophylactic agents introduces a different set of patient-related adverse events. Vancomycin, for example, has been associated with increased rates of postoperative acute kidney injury (AKI) in patients without underlying kidney disease when compared with β-lactam prophylaxis.^33^ Patients undergoing cancer-related reconstruction may be at higher risk due to prior chemotherapy exposure and older age, making the potential nephrotoxicity of second-line agents an important consideration.

In addition, disruption of the normal microbiome may paradoxically increase the risk of secondary infections. Certain antibiotics alter the gut flora, predisposing patients to *Clostridium difficile* infection (CDI).^34^ Clindamycin, a commonly used alternative in penicillin-allergic patients, has been consistently associated with higher risk of CDI. A study comparing doxycycline to clindamycin and cefdinir demonstrated significantly higher rates of CDI among patients with clindamycin use (aOR, 8.81; 95% Cl, 7.76 to 10.00) compared with cephalosporin (aOR, 5.86; 95% CI, 5.03 to 6.83).^35^ Although cephalosporin use may also increase CDI risk, this effect is more pronounced with later-generation cephalosporins rather than those typically used for surgical prophylaxis. First generation cephalosporins have been demonstrated to be associated with lower risk (aOR 2.88; 95% CI, 2.74–3.02) in contrast to their third generation counterparts (aOR 11.02; 95% Cl, 10.39 - 11.69). This extends beyond C. diff infections as a study looking at patients labeled as penicillin allergic had 23.4% more cases of *C. difficile* infection, 14.1% more MRSA infection and 30.1% more VRE infection.^27^^,^^36^

The consequences of second-line prophylaxis stretch beyond individual patient outcomes. The inappropriate use of broad-spectrum alternatives contributes to antimicrobial resistance.^37^ Cefazolin remains the preferred agent for surgical prophylaxis, as it provides broad coverage against common skin flora, including methicillin-susceptible *Staphylococcus aureus* (MSSA) and *Streptococcus* species, which predominate as primary pathogens in clean-contaminated procedures, while minimizing unnecessary broad-spectrum exposure.^38^ In contrast, patients labeled as penicillin allergic are often administered alternative agents such as vancomycin. Although active against methicillin-resistant *S. aureus* (MRSA), Vancomycin has poor activity against streptococci and no gram-negative coverage.^37^ Increased exposure to these alternatives has been associated with rising rates of MRSA infections and the emergence of vancomycin-resistant *S. aureus* (VRSA). While overall incidence remains low, VRSA rates in the United States have doubled since 2010.^39^^,^^40^

These downstream effects translate into significant healthcare utilization and cost burden. Patients receiving alternatives to cephalosporins have been shown to experience longer hospital stays, with an increase of 0.59 days in one study.^27^ The cumulative cost of these additional days was 9.5-fold higher compared to the cost of formal penicillin allergy testing for each patient ($64,626,630.48 vs $6776,327.340).^41^

Use of second-line agents is also associated with worse clinical outcomes that further increase healthcare burden. In arthroplasty patients, vancomycin as sole prophylaxis was associated with higher SSIs rates compared with patients who received cephalosporins,^42^ and a fourfold increase of SSIs has been reported in penicillin-allergic labeled patients receiving clindamycin for head and neck procedures.

Furthermore, antibiotic resistance (AMR) in this population is substantially higher, including vancomycin-resistant enterococci (VRE) with increased vancomycin use and *Clostridium difficile* infection with clindamycin, as discussed above.^27^ These complications lead to prolonged hospital stay, extended antibiotic therapy, and increased incidences of surgical revision.^37^ Prolonged hospital stay itself further compounds this risk, as increased length of stay has been associated with higher rates of MRSA infections in patients labeled as penicillin allergic, creating a reinforcing cycle of adverse outcomes.

Direct medication costs further compound this burden. A study investigating prophylaxis costs in total hip arthroplasty patients found that the cost of vancomycin prophylaxis was on average 2.6-fold higher than cefazolin prophylaxis. For total hip arthroplasty alone, the annual cost difference between these medications is estimated to sum to $2.4billion by 2040.^43^ Collectively, inappropriate use of alternative SAPs inadvertently results in higher costs of care, with current expenditures for resistant infections exceeding $4.6 billion annually and continuing to rise.^44^

## Conclusion

Review of the literature across a wide range of procedures supports cefazolin’s efficacy as a surgical prophylactic antibiotic. While there are not currently set protocols in place to guide antibiotic prophylaxis selection in plastic surgery, we strongly advocate for the use of cefazolin in a broad range of operations, unless a true contraindication is documented. Our own institution utilizes cefazolin as prophylaxis for numerous procedures due to its proven efficacy. However, due to 1.) common misreporting of penicillin allergies in patient charts and 2.) widespread misconceptions on allergic crossreactivity to all β-lactam antibiotics in penicillin-allergic patients, cefazolin is currently underutilized for prophylaxis in the surgical community. The subsequent use of second line prophylactic antibiotics can be detrimental to patient outcomes; use of these agents are associated with increased SSI rates, longer hospital stays, elevated cost of overall treatment, and increased risk of other adverse events (i.e. *Clostridium difficile* infection with clindamycin use, or AKI with vancomycin use). Increased provider education on the safety of cefazolin in the majority of the patient population, even those with history of rash and anaphylaxis to penicillin, is therefore vital to improving patients’ surgical recovery.

## Ethical approval

Ethical approval from a research ethics committee was not required as this study did not involve people, medical records, or anonymized human tissues.

## Funding

The authors received no financial support for the research, authorship, and/or publication of this article.

## Declaration of competing interest

The authors declare that they have no conflicts of interest to disclose.
